# A lightweight convolutional neural network for assessing an EEG risk marker for sudden unexpected death in epilepsy

**DOI:** 10.1186/s12911-020-01310-y

**Published:** 2020-12-24

**Authors:** Cong Zhu, Yejin Kim, Xiaoqian Jiang, Samden Lhatoo, Hampson Jaison, Guo-Qiang Zhang

**Affiliations:** 1 Department of Epidemiology, Human Genetics and Environmental Sciences, School of Public Health, University of Texas Health Science Center at Houston, Houston, TX USA; 2 School of Biomedical Informatics, University of Texas Health Science Center at Houston, Houston, TX USA; 3 Department of Neurology, McGovern Medical School, University of Texas Health Science Center at Houston, Houston, TX USA

**Keywords:** Convolutional neural network, Sudden death in epilepsy, PGES, EEG suppression, Deep learning

## Abstract

**Background:**

Convolutional neural network (CNN) has achieved state-of-art performance in many electroencephalogram (EEG) related studies. However, the application of CNN in prediction of risk factors for sudden unexpected death in epilepsy (SUDEP) remains as an underexplored area. It is unclear how the trade-off between computation cost and prediction power varies with changes in the complexity and depth of neural nets.

**Methods:**

The purpose of this study was to explore the feasibility of using a lightweight CNN to predict SUDEP. A total of 170 patients were included in the analyses. The CNN model was trained using clips with 10-s signals sampled from the original EEG. We implemented Hann function to smooth the raw EEG signal and evaluated its effect by choosing different strength of denoising filter. In addition, we experimented two variations of the proposed model: (1) converting EEG input into an “RGB” format to address EEG channels underlying spatial correlation and (2) incorporating residual network (ResNet) into the bottle neck position of the proposed structure of baseline CNN.

**Results:**

The proposed baseline CNN model with lightweight architecture achieved the best AUC of 0.72. A moderate noise removal step facilitated the training of CNN model by ensuring stability of performance. We did not observe further improvement in model’s accuracy by increasing the strength of denoising filter.

**Conclusion:**

Post-seizure slow activity in EEG is a potential marker for SUDEP, our proposed lightweight architecture of CNN achieved satisfying trade-off between efficiently identifying such biomarker and computational cost. It also has a flexible interface to be integrated with different variations in structure leaving room for further improvement of the model’s performance in automating EEG signal annotation.

## Background

Sudden unexpected death in epilepsy (SUDEP) is one of the leading causes (2.2–10 incidences per 1000 epilepsy patients) of death related to epilepsy [1–3]. Significant efforts have been dedicated to exploring efficient approaches to understand SUDEP risk factors in many recent studies. It was found that postictal generalized EEG suppression (PGES), which occurs 16–90% of generalized convulsive seizures (GCS) [4–8], is a marker of risk for sudden and unexpected death in epilepsy (SUDEP) [9]. However, the current manual review method in the clinical setting requires extensive labor from experienced domain experts and sometimes produce inconsistent results. In addition, motion and muscles effects inevitably adds noises to EEG signal making the identification of PGES even more challenging. Therefore it is important to develop an automatic method to assist the PGES annotation process.

Convolutional neural network (CNN) has been widely deployed in the image classification, segmentation and object detection tasks. It has gained popularity and achieved state-of-art performance in tasks related to the epileptic seizure detection in recent years [10–14]. However the application of CNN for identification of slow activity following generalized tonic-clonic (GTC) seizure remains as an underexplored area. In addition, limited effort has been spent on studying how noises in EEG signals impact a model’s prediction power in the CNN setting.

The purpose of this study is to develop an efficient CNN architecture to detect the onset of slow activity after a GTC seizure. At the same time we explored some variation in processing the input format of EEG and architecture of the proposed CNN model to understand their effect in model’s performance. In addition, we investigated the effect of noise from EEG’s signal on prediction accuracy by applying signal denoising functions.

## Methods and materials

### Data source

This work wasis based on a cohort of 170 patients with EEG recording from the 10 pairwise signal output from 13 electrodes (Fp1, F7, T7, P7, O1, Fp2, F8, T8, P8, O2, Fz, Cz, Pz). The EEG signals were resampled at a frequency of 200 Hz. A total of 134 patients were split out for the training of the model. This cohort was further randomly divided into 80 (60%) patients for internal training of the model and 54 (40%) patients for internal validation. The length of the signal in the training set varied from 2884 time points to 35,978 time points and each of these patients contains 10 s (10*200 Hz time points) length of slow activity signal. Clips with length of 10 s were cropped from the original EEG signal of these patients. Details of the method is discussed in the subsequent section. Thirty-six patients were held out as external testing set for the final evaluation of model’s performance. 12,345 snippets were generated from the original EEG signal of these 36 patients using a length of 10 s sliding window at every 0.1 s. The sampling yielded 26% of positive clips.

### Denoising EEG signal

EEG signal demonstrates a random pattern with varying levels of noises. Ideally, a prediction model would expect a “clean” input data with sufficient variance to facilitate its learning. Therefore we investigated whether such random noise from the EEG signal could raise difficulty for training CNN models (e.g. impaired accuracy). In order to explore the answer for this potential issue, we trained the prediction model using signal smoothed by the Hann function (1).

1 $$\begin{aligned} w_{0}(x)= & {} 0.5 \left( 1 - \cos \left( 2\pi \frac{n}{N}\right) \right) , 0<n \le N \end{aligned}$$

The level of denoising effect is controlled by the window length of this function. The wider the window is, the more noise will be removed. We selected window length of 5, 11 and 15 empirically in order to generate processed signals that visually demonstrated sufficient differences in the level of smoothness. Both raw EEG signal and denoised ones were used for training models

### Data augmentation of the training and validation set

**Fig. 1:**
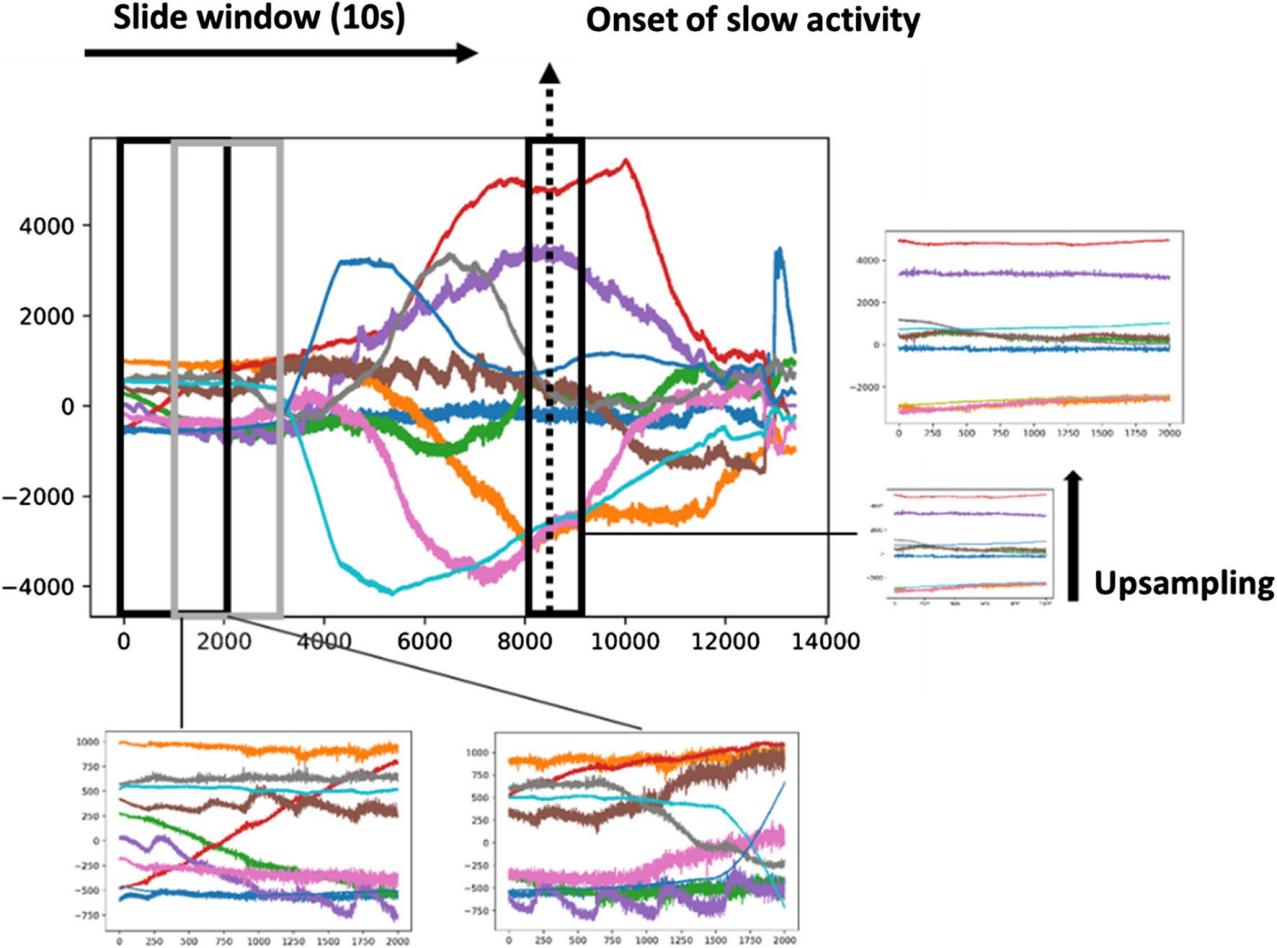
Crop clips of signals from raw EEG data. Bottom: clips with window length of 10 s; Right top: short clips that were cropped with window length of 5 s and were then up-sampled into 10 s

Since a significant proportion of our EEG signals is negative (non-slow-activity), training the model based on the imbalanced data could increase model’s risk of making false negative predictions. To mitigate this potential issue, we performed two augmentation approaches (Fig. 1) to boost the positive sample size during the training step.

The first method is cropping a 10 s clip from the original EEG signal using a sliding window. Instead of generating complete “clean” samples, we improved variation in the pattern of EEG signal by artificially sampling some clips that were mixed with both positive and negative signals. A signal is considered as being positive once it contains more than 50% (5 s) of positive signal and vice versa for the negative clips. The detailed description is as follow:

1. To crop positive clips, the cropping window would sliding from a pre-set starting point to the onset of slow activity. If the time gap between the beginning of EEG recording and onset of slow activity is less than 1000 Hz, the cropping started the time 0 of the original EEG recording. If the time gap is more than 1000 Hz, cropping starts at 950 Hz before the onset of slow activity.
2. To crop negative clips, the time gap between the beginning of EEG recording and onset of slow activity has to be more than 1000 Hz. The cropping window slide from the starting point of EEG recording till 1100 Hz before the onset of slow activity.

The second data augmentation approach is resampling short clips of signals around the area where slow activity occurs. The short clip is half-length (5 s or 1000 Hz) as the regular cropped clips from method 1, and will be upsampled to normal size using nearest-neighbor interpolation approach [15]. Signals near the changing point tended to be ambiguous and challenging for manual annotation. Therefore resampling clips around this area might potentially reinforce model’s learning in signal’s transition between being positive and negative, thus potentially benefit model’s discriminative power. A detailed description of this Sampling method is as below:

1. To crop the positive clips, the clips must contain negative signals longer than 5 s to be eligible for sampling. For the eligible clips, the cropping window would slide from a pre-set start point until 520 Hz before onset of slow activity. If the onset occurred 1000 Hz after EEG recordings began, the cropping started at time 0 the original EEG recording. If the time gap was more than 1000 Hz, the cropping starts at 1000 Hz before the onset of slow activity.
2. To crop negative clips, the cropping window slide between the onset of slow activity and 490 Hz before it occurred.

In a word, the both data augmentation procedures applied a slide window to ensure the sampling were performed to all electrodes’ signal at the same time so as to ensure the original spatial correlation among them. The sampled clips were then split into 60% training set and 40% internal testing set using random sampling scheme. In addition, all the clips were transformed into $10 \times 2000 \times 1$ dimensional arrays before feeding into the model.

### Development of the model

#### Architecture of the CNN

We designed a CNN with light architecture inspired by several successful examples from prior works [12–14]. We hypothesized that a lightweight structured CNN could achieve a similar level of accuracy in EEG related annotation task as the deep neural nets. The shallow architecture is more computational efficient and ideal for scenarios that require real-time monitoring and annotation of EEG signals.

**Fig. 2:**
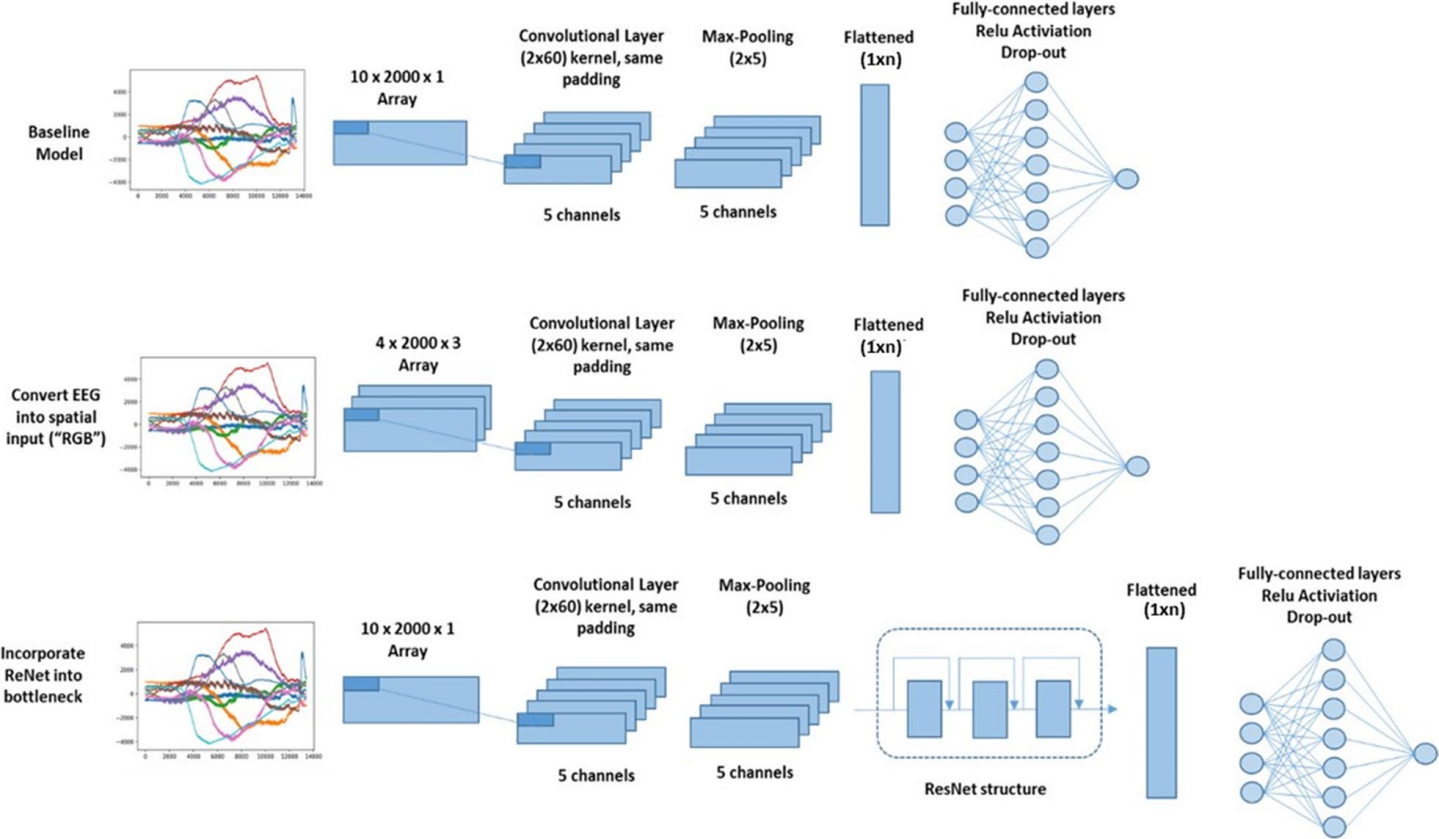
Architecture of the proposed CNN model

As shown by the Fig. 2 (baseline model), the first layer of the CNN model was implemented with 5 convolutional filters using size of 2*60. Specifically, it means that each individual filter will process 2 input channels with 60 time points of signal at one time. Convolutional filters would slide through all the signals at stride size of 1*60; the filters would perform computation on the signals inside the slide window and stride by every 60 Hz horizontally. Such process allows the CNN to analyze the underlying spatial or neurological correlations across all signal channels.

The convolutional layer was followed by an average pooling layer with filter sized at 5 by 5 dimension and strides size of 5 by 5 to reduce the output features from the convolutional layer. The purpose of the layer was to condense and generalize the processed information from the previous layers and reduce the learning effort for the CNN model.

The outputs of the average pooling layer were then flattened into 1 by n dimension in order to be fully connected to the subsequent dense layer with 20 neurons. Finally, a sigmoid activation function was implemented at the end of the CNN model for classification. Rectified linear unit (Relu) activation function was implemented across all the layers to speed up the training process. We also applied batch normalization to facilitate the optimization progress.

We also explored two variations in architectures of the model (Fig. 2). The first one was to convert the input structure of EEG signal array of shape 4*2000*3. Four pair-wise electrodes signals (fp1-f7, f7-t7, t7-p7, p7-o1; fz-cz, cz-pz, fz-cz, cz-pz; fp2-f8, f8-t8, t8-p8, p8-o2) were stored in 3 separated channels similar to the format of RGB image. CNN model could potentially benefit from such an approach regarding addressing the spatial correlations among the electrodes.

The second one is to incorporate residual network (ResNet) structure into bottle-neck position of the proposed baseline CNN [15]. ResNet consisted with a skipping architecture which allows for passing raw information at the beginning to deeper layers. It helps to solve the gradient vanish or explosion problems which cause trouble for convergence when neural nets attempt to go deeper in order to learn higher level features from the data.

#### Optimization of the hyperparamters of the CNN model

We fine-tuned all the hyper-parameters of the model empirically until the trend of loss function of both training set and testing set shows similar declining pattern without significant gap throughout the training epochs. To reduce potential overfitting issue, we applied drop-out [16] with rate at 0.5 to the fully connected layers after the flattened layer and dense layers right before the sigmoid activation function. We also applied additional regularizing methods including adding Gaussian noise to the flattened layer with standard deviation of 0.01, and l2 kernel regularizer with lambda of 0.01 to the dense layer. We chose stochastic gradient descent (SGD) optimizer with learning rate = 0.05, decay rate = 5e−6, momentum = 0.9 [17]. We also implemented Adam optimizer to examine its effect on the model performance [18]. The model was trained using a batch size of 64. To accelerate the training process, layers input was normalized using batch normalization technique [19].

#### Evaluation of the model

The evaluation of the testing set was based on the receiver operating characteristics (ROC) curve only. The reason is the distribution of positive and negative samples are artificially balanced across training and internal validation set, a significant proportion of EEG signal is slow-activity negative in the real world scenario; ROC curve, which assesses model’s diagnostic ability at varied discrimination threshold, is less sensitive to the imbalanced distribution in label and provides a more objective assessment of model’s performance across data with varied distribution. The specific evaluation was based on the area under the ROC curve (AUC).

## Results

**Fig. 3:**
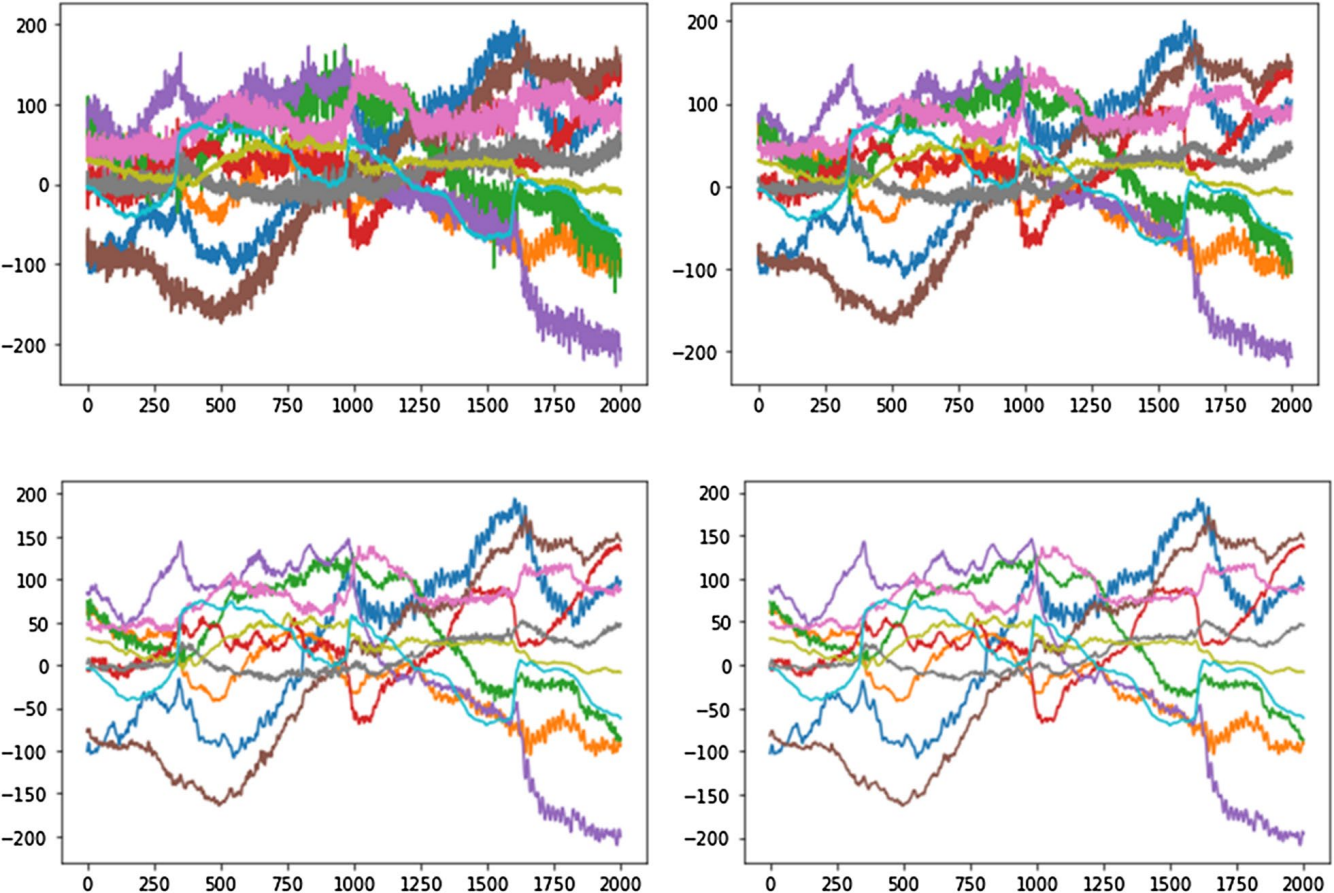
EEG signals before (left) and after (right) applying Hann smoothing function. Top left: raw signal; top right: window length = 5; bottom left: window length = 11; bottom right: window length = 15

Figure 3 compared the effect of smoothing function with varied window length (5, 11, and 15) in denoising the EEG signals. As shown by the figure, the Hann function significantly remove high frequency noise from the raw EEG signal. The filtered signals demonstrate a much “cleaner” pattern while retaining its original temporal shape. In addition, the denoising effect increased with expansion of window width without significantly distort signal’s characteristics.

Table 1 summarizes models’ best AUC using different structures and input signals after applying different levels of denoising filter. Interestingly, the proposed lightweight architecture achieved the best external testing-set AUC at 0.72 using raw signal optimized by Adam. Among the same models that were trained using filtered EEG, the best AUC (0.71) was achieved with a smooth window of length 3. The model’s performance did not seem to improve by increasing the window length of the smoothing function. In contrast to our expectation, the performance worsened after increasing the window length to 11 though recovered modestly to an AUC of 0.68. Of note, although the model that was trained with raw EEG signal achieved the best performance, the further stability test showed that raw EEG signal yielded more fluctuations in overall performance using either Adam or SGD optimizer than denoised signals. In general, smoothed EEG appeared yielded more reliable trained models according to our observance.

**Table 1:** Model performance using different level of denoised EEG signal

	Window length (none)	Window length (5)	Window length (11)	Window length (15)
Best AUC (SD)	0.72 (0.03)	0.71 (0.01)	0.66 (0.02)	0.68 (0.02)

As shown by the Table 2, the modification of part of the proposed baseline CNN architecture did not yield improvement in prediction power. The “RGB” channel format achieved slightly lower AUC at 0.7. Surprisingly, the incorporation of ResNet structure significantly impaired model’s performance. In terms of computation cost, the proposed baseline CNN architecture and one with with “RGB” format observed the least training time per epoch. The ResNet architecture took the longest training time which is 43–55 s per epoch. It took similar time of predicting the testing set (12,345 snippets) for baseline and baseline + ResNet architectures which is 4.4s and 4.8s respectively. The “RGB” structure observed the longest predicting time.

**Table 2:** The performance of the proposed model with variation in architecture

	Baseline	Baseline + “RGB” channel	Baseline + ResNet
Best AUC	0.72	0.7	0.56
Training time/ epoch	16–18 s	14–23 s	43–55 s
Prediction time (testing set)	4.4 s	11.17 s	4.8 s

In the further investigation of EEG channel’s contribution to CNN model’s overall prediction power, we focused on the baseline CNN architecture since it observed the best performance so far. We trained the same model using half of the EEG channels only at one time and evaluated the AUC of each training. As shown by the Table 3, the model with 1st half EEG channels (fp1-f7, f7-t7, t7-p7, p7-o1, fz-cz, cz-pz) achieved AUC of 0.634, while the one with second half EEG channels (fp2-f8, f8-t8, t8-p8, p8-o2, fz-cz, cz-pz) achieved AUC of 0.649.

**Table 3:** The performance of the proposed model with different input EEG

	Baseline	Baseline: 1st half EEG	Baseline: 2nd half EEG
Best AUC	0.72	0.63	0.65

## Discussion

In this study, we sought to explore the feasibility of using a light weight architecture of CNN model to identify the onset of slow activity from EEG signals. While deep structure could potentially advance neural network’s accuracy by letting it learn higher level features from the data, it remains questionable that whether a pay for more computationally complex solutions will yield an equal improvement in the model’s prediction power [20]. In addition, EEG signals that were obtained from different patient cohorts or monitoring machines could demonstrate varying underlying characteristics which affect the consistency in the model’s performance. Fine-tuning complex deep neural nets thus could be a tedious work. Therefore, it might be desirable to devise a lightweight architecture could make CNN more efficient in retraining and adapt to tasks that requires real-time action (e.g. EEG annotation, SUDEP prediction).

We developed a light-weight CNN architecture that required less computation time than complex ones. It took less than 5 s for the compact CNN model to classify 12345 snippets of EEG signals. This result demonstrated a potential of adopting well-trained CNN model to perform real-time monitoring tasks. The future direction of this work will involve evaluating the model in the online-environment. The proposed baseline model is also compatible with different variations in the architecture in case the tasks require increased complexities for the model. In searching the optimal CNN architecture, we failed to improve the proposed baseline model’s performance by bringing more complexities into the structure. By converting the input EEG data into an “RGB” format, we improved the training efficiency and potentially advanced model’s ability in addressing the underlying spatial correlations among the 13 electrodes. Yet such an approach did not yield an improved prediction power from the model. In contrary to our expectation, the integration of ResNet with the main architecture of the baseline model significantly worsened its performance despite of many successful examples from prior works. However, it should be noted that we didn’t re-optimize the hyper-parameters (number of layers, dropout rate etc.) of the model after applying these variations. Therefore the results should be interpreted with caution and further efforts are needed to explore a better way to assimilate these variations with the proposed CNN model with light architecture.

We also investigated CNN model’s sensitivity to the noise in the signal. Surprisingly we found that the model used raw EEG signal achieved the best performance of AUC at 0.72. The same model that used lightly filtered signal observed comparable AUC at 0.71. However, by implementing a more aggressive denoising filter, the progress became counterproductive which might be resulted from the loss of information. A further investigation found that models that were trained with raw EEG signal yielded less stable results than those using filtered signals. This result indicated that a moderate amount of random noise from the signal might contain essential information for training reliable prediction model. However the denoising progress should be implemented with caution as it might sacrifice some underlying key information for ensuring a reliable model. It requires trials and errors to identify a balance between noise removal and model efficiency.

There are several limitations in this study. The evaluation of models’ performance regarding its computational cost and prediction power were implemented on the same cohort of patients although models were fine-tuned based on the training and validation sets and assessed using a separate testing set. In order to comprehensively exam the performance of proposed models, future work will require using independent external data. When exploring the effect of denoising EEG on model’s overall prediction power, we implemented a Hann’s function with arbitrarily selected window size (5, 11, 15) to created EEG signals. These values were selected empirically to ensure a straightforward study of the potential correlation between signals’ visual smoothness and models’ prediction power. Such as approach however more remove essential variations in the information inside the signal. Therefore future work demands a more robust denoising method such as autoencoders.

## Conclusion

Post-seizure slow activity in EEG is a potential marker for SUDEP. Our proposed light weight architecture of CNN achieved satisfying performance in identifying such marker with less computational cost than deep neural nets. Our model has a flexible interface to be integrated with different variations in structure which leaves room for further enhancing model’s performance. We found the model’s accuracy was dependent on the quality of the EEG signal. However, the over-cleaned data did not guarantee an improvement in prediction power. In future work, we will incorporate more robust denoising technique and improve the integration of complex CNN structure with the proposed model.

## Data Availability

The data include protected health information, thus are not publicly available.

## Abbreviations

CNN: Convolutional neural network
EEG: Electroencephalogram
SUDEP: Sudden unexpected death inepilepsy
ResNet: Residual network
PGES: Postictal generalizedEEG suppression
GCS: Generalized convulsive seizures
GTC: Generalized tonic-clonic
SGD: Stochastic gradient descent
ROC: Receiver operating characteristics
AUC: Area under the ROC curve
